# A Lesson in Self-Experimentation

**Published:** 1913-11-15

**Authors:** 


### A LESSON IN SELF-EXPERIMENTATION.

A FEW years ago Dr. Henry Head submitted himself to the division of one of the nerves of his arm in order that he might make reliable observations on the progress of the return of function while the nerve regenerated. As was noted at the time in THE HOSPITAL, this distinguished neurologist's observations on his own vivisected body threw much new light upon the whole mechanism of sensation and the sensory nerves. From them Dr. Head built up a hypothesis of two distinct varieties of afferent nerves—the nerves, that is to say, which carry sensations *to* the brain, as opposed to those which transmit to the muscles the orders sent out *from* the brain. These two kinds of sensibility he termed "protopathic" and "epicritic" respectively; the former concerned especially with the perception of pain, great heat, and great cold; the latter with tactile sensations other than painful and with the finer degrees of variation in temperature.

Since 1907 two workers at University College Hospital, Messrs. Wilfred Trotter and H. M. Davies, have been engaged in similar but more extensive auto-vivisection experiments; and the results, which have been published in a German periodical (*Journal f. Psychol. und Neurol.*) and summarised in the *Lancet*, are somewhat at variance with those of Dr. Head. After investigations of the regrowth of several cut nerves in their own bodies, a process which takes months or years of time, they doubt whether the subdivision of sensation into the protopathic and epicritic systems can be strictly maintained. For they found a good deal of evidence pointing to both sorts of function being possible in one and the same nerve. When a cut nerve is regenerating all forms of sensation tend to return together.

The issue of this conflict of opinion on a highly technical and scientific matter must be left in the hands of experts in neurology. But institutional workers, whether medical men or not, can appreciate the courage and self-devotion of the two University College surgeons and the London Hospital physician, who have thus vivisected themselves (or each other) in the "cause of science." None of the three scientists, we feel sure, wants to pose as having done anything heroic or extraordinary. A pure zeal for the advancement of knowledge may reasonably be presumed to have been their sole motive.

